# The Employment of Collodion and Asbestos in Plugging Teeth

**Published:** 1849-07

**Authors:** 


*The Employment of Collodion and Asbestos in Plugging Teeth.*—Mr. Robinson in a communication to the London Lancet, says: I have frequently applied collodion in severe cases of tooth-ache, arising from exposure of the nerve, with perfect success, when no persuasion could induce the patient to submit to extraction, either with or without the use of chloroform or ether. The method I adopt is, to let the patient first wash the mouth with warm water, in which a few grains of bicarbonate of soda have been dissolved. I then remove from the cavity any foreign substance likely to cause irritation. After drying the cavity, I drop from a point the collodion, to which have been added a few grains of morphia, after which I fill the cavity with asbestos, and saturate with collodion. Lastly, over this I place a pledget of bibulous paper. In a few seconds the whole becomes solidified, and forms an excellent non-conductor of heat and cold to the exposed nerve. By occasionally renewing this I have been enabled to effect a more durable stopping with gold leaf.

